# Identifying Important Nodes in Complex Networks Based on Node Propagation Entropy

**DOI:** 10.3390/e24020275

**Published:** 2022-02-14

**Authors:** Yong Yu, Biao Zhou, Linjie Chen, Tao Gao, Jinzhuo Liu

**Affiliations:** 1 School of Software, Yunnan University, Kunming 650091, China; yuyong@ynu.edu.cn (Y.Y.); zhou98@mail.ynu.edu.cn (B.Z.); 12019101059@mail.ynu.edu.cn (L.C.); 2 Key Laboratory in Software Engineering of Yunnan Province, Yunnan University, Kunming 650091, China; 3 School of Education, Yunnan University of Business Management, Kunming 650033, China

**Keywords:** complex networks, important nodes, node propagation entropy, epidemic models, importance metric

## Abstract

In recent years, the identification of the essential nodes in complex networks has attracted significant attention because of their theoretical and practical significance in many applications, such as preventing and controlling epidemic diseases and discovering essential proteins. Several importance measures have been proposed from diverse perspectives to identify crucial nodes more accurately. In this paper, we propose a novel importance metric called node propagation entropy, which uses a combination of the clustering coefficients of nodes and the influence of the first- and second-order neighbor numbers on node importance to identify essential nodes from an entropy perspective while considering the local and global information of the network. Furthermore, the susceptible–infected–removed and susceptible–infected–removed–susceptible epidemic models along with the Kendall coefficient are used to reveal the relevant correlations among the various importance measures. The results of experiments conducted on several real networks from different domains show that the proposed metric is more accurate and stable in identifying significant nodes than many existing techniques, including degree centrality, betweenness centrality, closeness centrality, eigenvector centrality, and H-index.

## 1. Introduction

Complex systems in many real-world domains are modeled as complex networks to ensure efficient analysis. Such systems include electrical networks [1], social networks [2,3], protein–DNA networks [4], transportation networks [5], and biological networks [6]. In recent years, the identification of the essential nodes in complex networks has attracted significant interest because of their important theoretical and practical significance, e.g., in preventing and controlling epidemic diseases [7,8], controlling the spread of rumors [9,10], proposing new marketing strategies [11], and discovering essential proteins [12]. Faced with a large-scale complex network, the question of how to rapidly and productively determine the important nodes in the network is the first challenge that has to be addressed in the study of node importance.

Several importance metrics have been proposed to evaluate the significance of nodes from a network topology perspective. They include degree centrality [13], betweenness centrality [14], closeness centrality [14], k-shell [15], and eigenvector centrality [16]. Degree centrality, which asserts that the more neighbors a node has, the more influential it is, is relatively simple, intuitive, and easy to implement. However, it generally does not consider the global properties of the network or the individual properties of nodes. Therefore, it does not perform well in most real-world applications. Betweenness centrality asserts that the more times the shortest length path between any pair of nodes in the network passes over one node, the more mediated the node is, and the more critical in the network. Closeness centrality asserts that the smaller the mean value of the shortest length path from a node to the rest of the nodes in the network, the more influential the node is in the network. K-shell asserts that the closer the node is to the core of the network, the more critical it is. Nodes within the same shell are considered to have the same importance and the same scalability. However, k-shell is unsuitable for real networks. Eigenvector centrality asserts that a node’s importance is determined by the number and importance of its neighbors (i.e., the degree of the node). However, it is unsuitable for large networks.

Zhao et al. [17] asserted that the significance of a node is related to both its own significance and to that of the nodes to which it connects. However, any metric that takes this into consideration will have high time complexity. Ullah et al. [18] proposed a Local and Global Centrality (LGC) measurement algorithm that identifies significant nodes by processing both local and global information about network topology. Methods have been proposed based on random walks, e.g., the famous PageRank [19], LeaderRank [20], VoteRank [21], and HITS [22]. Entropy has been used to scale the importance of nodes as well [23,24]. For example, Zareie et al. [25] proposed a method based on information entropy to test the propagation ability of a node in a network according to the topological information of the node. Fei et al. [26] integrated the advantages of existing importance metrics and proposed a method to identify critical nodes using relative entropy and the technique for order performance by similarity to idea solution (TOPSIS) method. Hu et al. [27] proposed a sorting method for influential nodes with structural holes. Thus, the use of entropy for identifying key nodes has a sound theoretical basis and is the basis of several proposed approaches.

The idea of complex networks originated in the field of statistical physics, where entropy is an important concept. The primary starting point of this study is the application of entropy to the identification of important nodes in complex networks. Traditionally, Shannon entropy [24] has been used to analyze the overall statistical characteristics of networks. The centrality of nodes considers the importance of a node in the whole network; thus, the entropy of nodes can be considered as reflecting the importance of a node. Furthermore, the decision tree, which is very common in machine learning for data analysis, is also based on entropy.

Inspired by the useful applications of entropy outlined above, we propose a novel entropy-based metric to measure the importance of nodes in complex networks. The proposed metric, called node propagation entropy, combines the clustering coefficients of nodes and the influence of the first- and second-order neighbor numbers on node importance from an entropy perspective. We evaluate the proposed node propagation entropy metric via epidemic models and robustness experiments. The results of experiments conducted on twelve real networks from different domains show that the proposed node propagation entropy metric provides superior performance in terms of monotonicity, rankings list accuracy, and stability. The application of node propagation entropy in complex networks expands the practical application of information entropy theory.

The remainder of this paper is structured as follows: Section 2 describes the critical metrics used in the comparison experiments; Section 3 provides the details of the proposed node propagation entropy metric and its implementation; Section 4 analyzes the experimental performance of the proposed metric; and Section 5 provides concluding remarks.

## 2. Centrality Indicators

A complex network can be modeled as *G* = (*V*,*E*), where *V* = {*v*_1_,*v*_2_,…,*v_n_*} is a set of nodes, *E =* {*e*_1_,*e*_2_,…,*e_m_*} is the set of edges between the nodes, and *n*, *m* denote the number of nodes and edges in the network, respectively. Each edge in *E* is located between its corresponding nodes in *V*.

Many methods have been proposed for the identification and determination of the significance of key nodes in complex networks in terms of degree centrality, betweenness centrality, closeness centrality, eigenvector centrality, H-index, and GIN. The definitions of these methods and metrics are as follows.

**Definition** **1** (Important Nodes)**.** *Important nodes are nodes that play an essential role in the organization of network structure or the dynamical behavior of network system [28]. Previous research has generally used node centrality to quantify node importance. We propose the node propagation entropy metric to quantify the importance of nodes by calculating node centrality from an entropy perspective.*

**Definition** **2** (Degree Centrality, DC)**.**
*The DC [13] of a node, i, is calculated as follows:*

(1) $$DC(i)=\sum_{j\neq i}^{n}a_{ij}$$

*where node j represents any node other than i, n is the total number of nodes, and* $a_{ij}$ *denotes the edge between nodes i and j. If an edge exists,* $a_{ij}$ *has a value of “1”; otherwise, its value is “0”.*

**Definition** **3** (Betweenness Centrality, BC)**.** *The BC [14] of a node, i, is calculated as follows:*

(2) $$BC(i)=\sum_{j,l\neq i}\frac{g_{jl}(i)}{g_{jl}}$$

*where nodes j and l represent any two nodes other than i, g_jl_ denotes the number of shortest length paths from node j to node l, and g_jl_(i) denotes the number of shortest length paths from node j to node l via node i.*

**Definition** **4** (Closeness Centrality, CC)**.** *The CC [14] of a node, i, is calculated as follows:*

(3) $$CC(i)={\left[\sum_{j}^{N}d_{ij}\right]}^{-1}$$

*where d_ij_ denotes the distance from node i to node j.*

**Definition** **5** (Eigenvector Centrality, EC)**.** *The EC [16] of a node, i, is evaluated as follows:*

(4) $$EC(i)=x_{i}=c\sum_{j\in N(i)}a_{ij}x_{j}$$

*where N(i) denotes the neighbors of node i, c is a constant, x_i_ signifies the importance of node i, and* $a_{ij}$ *denotes the edge between nodes i and j. If the edge exists, then* $a_{ij}$ *has a value of “1”; otherwise, its value is “0”. We denote* $x={\left[x_{1},x_{2},x_{3},\ldots ,x_{n}\right]}^{T}$ *, and after several iterations to reach the steady-state, we obtain the following form:*

(5) x=cAx

*where x is an eigenvector corresponding to the eigenvalue c* ^−1^ *of matrix A. In addition, it can be expressed as* $Ax=c^{-1}x\;$ *, where* $c^{-1}$ *is the eigenvalue of matrix A.*

**Definition** **6** (H-index)**.** *Lü et al. [29] constructed the operator H to act on a limited number of real numbers (x_1_,x_2_,…,x_n_) which return an integer y, where y is the maximum value satisfying the condition that there are at least y items in (x_1_,x_2_,…,x_n_), each not smaller than y. Then, the H-index of node i is calculated as follows:*

(6) $$h_{i}=H(k_{a},k_{b},\ldots ,k_{c})$$

*where k_i_ denotes the degree of node i and* $k_{a},k_{b},\ldots ,k_{c}$ *denote the degrees of the node’s neighbors. We define h_i_^(0)^ = k_i_ to be a node i with zero-order H-index. More generally, an n-order H-index (n > 0) is defined as follows:*

(7) $${h_{i}}^{\left(n\right)}=H({h_{a}}^{\left(n-1\right)},{h_{b}}^{\left(n-1\right)},\ldots ,{h_{c}}^{\left(n-1\right)})$$

*where the first-order H-index value is the final H-index value, i.e., h_i_^(1)^ = h_i_.*

**Definition** **7** (GIN)**.** *The GIN [17] of a node, i, is calculated as follows:*

(8) $$GIN(i)=e^{\frac{k_{i}}{n}}\sum_{i\neq j}\frac{k_{j}}{d_{ij}}$$

where k_i_ denotes the degree of node i and * signifies the multiplication operator.

**Definition** **8** (LGC)**.** *The LGC [18] of a node, i, is calculated as follows:*

(9) $$LGC(i)=\frac{k_{i}}{n}\sum_{j\neq i}\frac{\sqrt{k_{j}+\partial }}{d_{ij}}$$

*where*∂*is a tunable parameter. LGC performs best when*∂=0.4*[18]; therefore, we herein set*∂=0.4.

## 3. Materials and Methods

In general, the importance of a node and its impact on other nodes are enhanced when the node effectively spreads information throughout the network. To evaluate the ability of nodes to locate important nodes in complex networks, we propose the node propagation entropy (*PE*) metric, which combines the clustering coefficients of nodes and the influence of the first- and second-order neighbor numbers on node importance from an entropy perspective.

### 3.1. Node Propagation Entropy

Nodes prefer to form relatively tightly connected groups with each other [30]. This tendency is greater than the average probability of establishing a random relationship between two nodes. In complex networks, the clustering coefficient is used as a measure of the degree of node clustering. The clustering coefficient is divided into the global and local clustering coefficients. This study uses the local clustering coefficient. The local clustering coefficient of a node *i* is given by the ratio of the actual number of edges existing between the neighboring nodes of node *i* to the largest number of possible edges. Thus, the local clustering coefficient of an undirected network is defined as follows:

(10) $$c_{i}=\frac{2\sum_{j,k\in N(i)}a_{jk}}{k_{i}(k_{i}-1)}$$

where *N*(*i*) denotes all first-order neighboring nodes of node *i* and *k_i_* denotes the degree of node *i*. If *k_i_* ≤ 1, we allow *c_i_* = 0.

Degree centrality states that any increase in a node’s neighbors extends its influence; therefore, we extend degree centrality to second-order neighbors. The propagation ability of a node is related to both the number of first-order neighbors and to the number of second-order neighbors, which is comparable to the probability of befriending the friend of a friend. Further, we consider the effect of the clustering coefficient on node propagation ability. According to the random walk theory, information from a node in the network is transmitted to other nodes to which it is connected with a certain probability; the larger the clustering coefficient of a node, the easier it is for the information to be transmitted back. Therefore, the larger the clustering coefficient, the worse the node’s propagation ability. The larger the average node clustering coefficient, the slower the propagation if other network parameters remain constant.

Accordingly, we propose the idea of Clustering Coefficient and Neighbors (*cn*), which describes the local propagation capacity of nodes and is defined as follows:

(11) $$cn_{i}=(N_{2}(i)+N(i))/(1+c_{i})$$

where *N*(*i*) and *N*_2_(*i*) represent the number of first- and second-order neighbors of node *i*, respectively; *c_i_* denotes the clustering coefficient of node *i*.

*cn* only considers local information, not global information. Entropy has been successfully used to assess the significance of nodes in a network [31]. Therefore, we adopt the definition of information entropy and propose node *PE*, which considers a certain amount of global information in the network as an indicator of node importance.

Node *PE* is defined as follows in Equations (12) and (13):

(12) $$I_{i}=\frac{cn_{i}}{\sum_{j=1}^{n}cn_{j}}$$

where node *j* denotes any node in a network, *n* is the total number of nodes, and *cn_j_* denotes the local propagation capacity of node *j*.

(13) $$PE_{i}=-\sum_{j\in N(i)}I_{j}\ln I_{j}$$

where *N*(*i*) denotes all first-order neighbors of node *i* and *j* represents any node in *N*(*i*).

Equation (12) compensates for the deficiency in Equation (11), where the local propagation capacity only considers local information about the nodes. More specifically, it considers a certain amount of global information about the network which replaces the relative importance of the nodes with the ratio of the local propagation capacity of each node to that of the total nodes.

Meanwhile, Equation (13) is based on the definition of information entropy considering the relative importance of nodes obtained from Equation (12). It replaces the probability of *j* with the relative importance of neighbor node *j* to derive the *PE* of node *i*. Node *PE* states that the importance of a node should be assessed by considering all its neighbors, and that each neighbor node contributes differently. $-I_{j}\ln I_{j}$ indicates the contribution of neighbor node *j*. The importance of node *i* is equal to the sum of the contributions of all neighbors of node *i*.

Algorithm 1 outlines the calculation process of *PE*.
**Algorithm 1:** Propagation entropy (*PE*) computation procedure**Input:** *G =* (*V*,*E*)1: Initialize network *G*2: **for each** vertex *i* in *V* **do**3: if *i* degree ≤ 1:4:  *c_i_* = 05: else:6:  compute *c_i_* via Equation (10)7: **end for**8: *sumcn = 0.0*9: **for each** vertex *i* in *V* **do**10: *sumneigh* = *N*(*i*) *+ N*_2_(*i*)11: *cn_i_* = *sumneigh*/(1 *+ c_i_*)12: *sumcn* += *cn_i_*13: **end for**14: **for each** vertex *i* in *V* **do**15: *I_i_* = *cn_i_/sumcn*16: **end for**17: **for each** vertex *i* in *V* **do**18: *sum_I_i_ = 0.0*19: **for each** vertex *j* in *N*(*i*) **do**20:  *sum_I_i_ +=* −*I_j_ln*(*I_j_*)21: *PE_i_* = *sum_I_i_*22: **end for**23: Rank the *PE* value of all nodes**Output:** An ordered list of nodes

The *PE* computation procedure comprises four steps. First, calculate the clustering coefficient of each node (time complexity: O(n^2^)). Second, calculate the number of second-order neighbors (time complexity: O(n^2^)). The time complexity of the third and fourth steps is O(n). Consequently, the aggregate time complexity of *PE* is O(n^2^).

### 3.2. Effectiveness of the Proposed Node Propagation Entropy Metric

In this section, the yeast protein interaction network of Saccharomyces cerevisiae [32] is used to verify the effectiveness of the proposed node *PE* metric. Nodes in the network represent proteins, while each edge represents the interactions between two proteins. The yeast–protein interaction network contains 5093 nodes and 24,743 edges. Of the 5093 nodes in the yeast network, 1167 are important, 3591 are unimportant, and the importance of the remaining 335 is unknown.

We used ten different centrality measures to identify important proteins in this yeast network. Further, we used the precision metric [33] to evaluate the accuracy with which the important proteins are identified. The indicator only considers whether the top *k* nodes are predicted accurately; its value is equal to the proportion of nodes in the first *k* nodes that are predicted accurately. The precision metric is defined as follows:

(14) $$precision=\frac{n_{p}}{k}$$

where *n_p_* denotes the number of important nodes contained in the first *k* nodes of the prediction. *k* was set as 1167 in this yeast network test.

It can be seen from Table 1 that the accuracy of the *PE* metric is on par with that of other importance measures, and *PE* can accurately identify important nodes in the network. These results indicate that it is reasonable and adequate to use *PE* as a metric for evaluating node importance.

## 4. Experiments and Results

### 4.1. Date

To better demonstrate the validity of the node *PE* metric for representing the importance of nodes, we evaluated it on twelve real networks from different domains. None of the networks allow for the existence of self-loops, i.e., two vertices of an edge having the same vertex. The twelve real networks comprised (i) two human social networks, the Train [34] and Karate [35] networks; (ii) a collaboration network, Ca_Sandi_Auth [36]; (iii) an animal network, Dolphins [34]; (iv) a DIMACS10 and a bio-c. elegans neural network [36]; (v) an email network, Email-Enron [36]; (vi) two miscellaneous networks, PolBooks [34] and AdjNoun [36]; (vii) an interaction network, Crime [34]; (viii) a metabolic network, Yeast [34]; a co-authorship network, Netscience [34]; and an infrastructure network, Uspowergrid [34]. These networks are publicly available and were downloaded from http://konect.cc/networks/ (accessed on 13 December 2021) and https://networkrepository.com/networks.php (accessed on 13 December 2021).

The topological features of the network dataset are presented in Table 2.

### 4.2. Evaluation of the Susceptible–Infected–Removed Model

**Figure 1:**
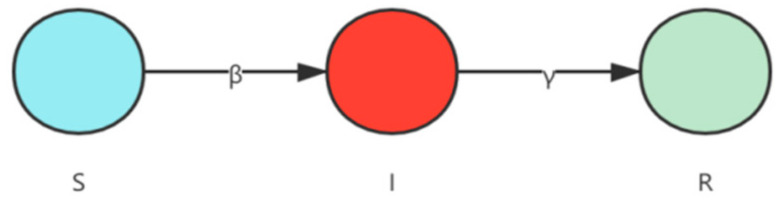
Susceptible–infected–removed (SIR) epidemic model.

This section focuses on the principles and implementation of the susceptible–infected–removed (SIR) model to set the stage for subsequent experiments. In the study of infectious disease dynamics, to analyze the influence of crucial nodes in complex networks, the SIR model [37] (developed by Kermack and McKendrick in 1927 when they studied the transmission patterns of the Black Death and plague) is frequently employed. The SIR model replicates the natural state of disease transmission. It divides the population into the following three categories: S for susceptible, I for infected, and R for removed (Figure 1).

The proportions of individuals in susceptible, infected, and recovered status as a percentage of the total over time is expressed as follows:

(15) $$\begin{array}{l} \frac{ds(t)}{dt}=-\beta i(t)s(t)\\ \frac{di(t)}{dt}=\beta i(t)s(t)-\gamma i(t)\\ \frac{dr(t)}{dt}=\gamma i(t) \end{array}$$

where, *s*(*t*), *i*(*t*), and *r*(*t*) denote those nodes in susceptible, infected, and recovered status at time *t*, respectively; *β* is the infection rate; and *γ* is the recovery rate.

**Figure 2:**
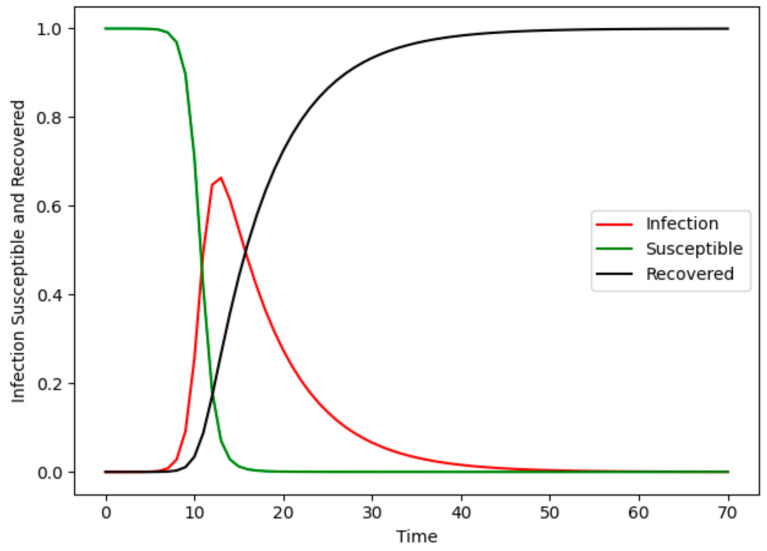
Variation in the percentage of different state nodes in the SIR model.

In the SIR model, the proportion of nodes in different states to all nodes varies with time (Figure 2). When the probability of infection is high, all susceptible nodes eventually become infected over time, whereas all infected individuals are eventually in the recovered state.

We applied the SIR model to analyze the impact of critical nodes in the complex networks and to model the propagation of information between the nodes. The propagation process was as follows. First, one node was infected, and all others were susceptible. In each time step, nodes in the infected condition infected other neighboring nodes in the susceptible condition with a probability *β* (here, set as the prevalence threshold of the network). Second, each of the previously infected nodes entered the recovered state with a probability *γ* (set as 1) [18,38]; nodes in the recovered state would not be reinfected. The process of propagation was reiterated until the network was free of infected nodes. The propagation capability of node *i* is expressed by *R_i_*, which is the mean number of final nodes recovered over 1000 independent runs, with each independent run of node *i* being the only infected seed. The higher the value of *R_i_*, the better is the propagation capability of node *i*. Third, a list of nodes ranked in descending order of importance was obtained based on the propagation ability of the nodes in the network.

Based on the theory of heterogeneous mean fields [39], the prevalence threshold of the SIR model was approximated as

(16) $$\beta_{c}=\frac{<k>}{<k^{2}>-<k>}$$

where *<k>* denotes the mean degree of community *c*.

### 4.3. Evaluation of the Susceptible–Infected–Removed–Susceptible Model

**Figure 3:**
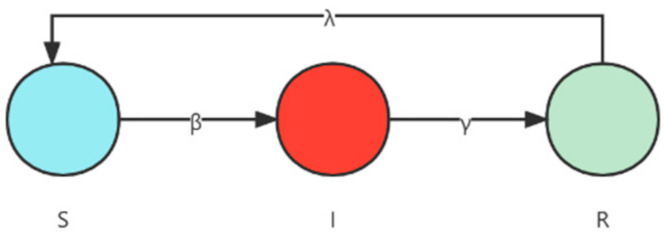
Susceptible–infected–removed–susceptible (SIRS) epidemic model.

The SIR model, the Susceptible–Infected–Removed–Susceptible (SIRS) model [40], is obtained by adding antibody time to the SIR model. Further, in the SIRS model the recovered only have temporary immunity. After the antibody time, they become susceptible and may be infected again. This divides the total population into the following three categories: S for susceptible, I for infected, and R for removed (Figure 3).

The proportions of individuals with susceptible, infected, and recovered status as a percentage of the total over time is expressed as follows:

(17) $$\begin{array}{l} \frac{ds(t)}{dt}=-\beta i(t)s(t)+\lambda r(t)\\ \frac{di(t)}{dt}=\beta i(t)s(t)-\gamma i(t)\\ \frac{dr(t)}{dt}=\gamma i(t)-\lambda r(t) \end{array}$$

where, *s*(*t*), *i*(*t*), and *r*(*t*) denote those nodes with susceptible, infected, and recovered status at time *t*, respectively; *β* is the infection rate; λ is the loss of immunization rate; and *γ* is the recovery rate.

### 4.4. Kendall Coefficient (τ)

We used the SIR and SIRS models to produce a descending order rankings list of node importance based on their propagation ability in the network. The Kendall coefficient, *τ* [41], was applied to estimate the correlation between the importance rankings list obtained by each importance measure and the real importance rankings list generated by the SIR model. The higher the *τ* value, the higher the correlation between the two rankings lists and the higher the accuracy of the results obtained by the method. The closer the Kendall coefficient is to one, the more accurate the ranking result is and the more effective the method is in identifying important nodes.

The Kendall coefficient considers a pair of binary groups consisting of two sets of random variables, *X* and *Y*. For any pair (*X_i_*, *Y_i_*) and (*X_j_*, *Y_j_*), the pair of binaries is said to be consistent if both *X_i_ > X_j_* and *Y_i_ > Y_j_* or *X_i_ < X_j_* and *Y_i_* < *Y_j_*. They are considered inconsistent if *X_i_ > X_j_* and *Y_i_ < Y_j_* or *X_i_ < X_j_* and *Y_i_* > *Y_j_*; if *X_i_* = *X_j_* or *Y_i_ = Y_j_*, the pair is neither consistent nor inconsistent. The Kendall coefficient, *τ*, is calculated as follows:

(18) $$\tau =\frac{2(n_{c}-n_{i})}{n(n-1)}$$

where *n_c_* and *n_i_* denote the number of consistent and inconsistent binary groups, respectively, and *n* denotes the number of binary groups. The Kendall coefficient *τ* is in the range [−1, 1]. Ideally, if *τ* = 1, then the rankings list produced by the degree centrality metric is identical to the rankings list produced by the actual propagation process.

### 4.5. Epidemic Models Experiment

This section compares the node *PE* metric with nine other node importance metrics: K-shell++, DC, BC, CC, EC, *PE*(N2), H-index, GIN, and LGC. In order to compare the performance of *cn* and *N*^2^, we use *PE* for *PE* calculated with *cn* and *PE*(*N2*) for *PE* calculated with *N*^2^. First, the SIR and SIRS models were applied to determine the impact of nodes on the dynamic propagation process to obtain the node propagation ability generated by the natural propagation process of the ranked list. Then, the Kendall coefficient was applied to estimate the extent to which the node *PE* metric was similar to the propagation capability of a single node. The performance of the other nine comparison metrics was similarly measured using the Kendall coefficient.

**Figure 4:**
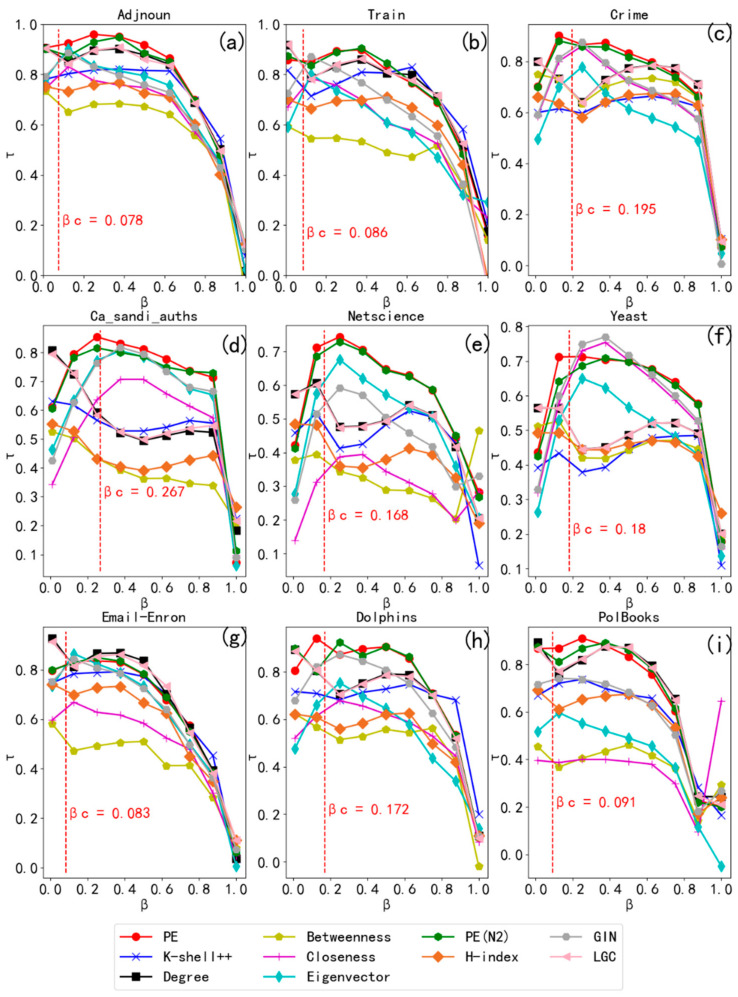
Performance comparison of the critical indicators in the SIR model for different values of *β* (infection rate). Each subgraph (**a**–**i**) of the graph represents a real network; the network name is at the top of the graph and the vertical dashed line inside the graph indicates the prevalence threshold of that network. The performance of the node propagation entropy, *PE*, is highlighted in red, and the performance of the node propagation entropy, *PE*(*N2*), is highlighted in green. The higher the *τ* value, the better the performance of the corresponding centrality methods.

The correlation between the rankings lists provided by the ten different importance measures for nine real networks in different domains and the rankings list obtained from the SIR model by adjusting the infection rate, *β*, are depicted in Figure 4.

Figure 4 shows that the fold of the node *PE* metric is at the top of each comparison plot, especially near the threshold value where the value of *τ* for *PE* is largest, indicating its effectiveness in identifying vital nodes. When 0.1 ≤ *β* ≤ 0.4, *PE* obtained larger *τ* values in the nine real networks, especially in the Adjnoun, Ca_Sandi_Authh, and PolBooks networks, indicating that *PE* more accurately identified important nodes in the networks. In large networks, such as Crime and NetScience, *PE* performed well and obtained the maximum *τ* value for both networks, indicating that *PE* has an advantage in identifying important nodes. In contrast to certain centrality measures, the performance of which fluctuated widely from network to network, the node *PE* metric performed well across networks, indicating its stability.

When the contagion probability, *β*, is very low in the SIR model, the disease does not spread because the infected node has only a small probability of infecting its neighbors; hence, the node only infects a limited area or not at all, making it difficult to measure the proper spread of the node. Conversely, when the transmission rate is high, the disease infects a large proportion of nodes regardless of which node it started from, which is meaningless for comparing the impact of individual nodes. Therefore, we focused on the range in which the transmission rate was around the epidemic threshold [39].

From Table 3, we obtained the *τ* values of each importance metric under the prevalence threshold of each network, from which it can be seen that the node *PE* metric performed better than the other importance metrics; *PE* performed best for eight of the nine networks.

The nine real networks used in the SIR model experiments are representative networks in various fields, and the experimental results are general. Figure 4 and Table 3 show that *PE* obtained higher *τ* values for all nine networks, especially the Ca_Sandi_Auth, Email-Enron, Dolphins, Crime, and Polbooks networks, for which the value was higher than that of the other nine methods. The closer *τ* was to one, the more accurate the sorting results were. We found that *PE* tends to perform better than *PE(N2)*, indicating that *cn* is better suited to identify important nodes than *N*^2^. The results show that *PE* identified the critical nodes in the network more accurately, and had strong applicability and good performance in most networks.

**Figure 5:**
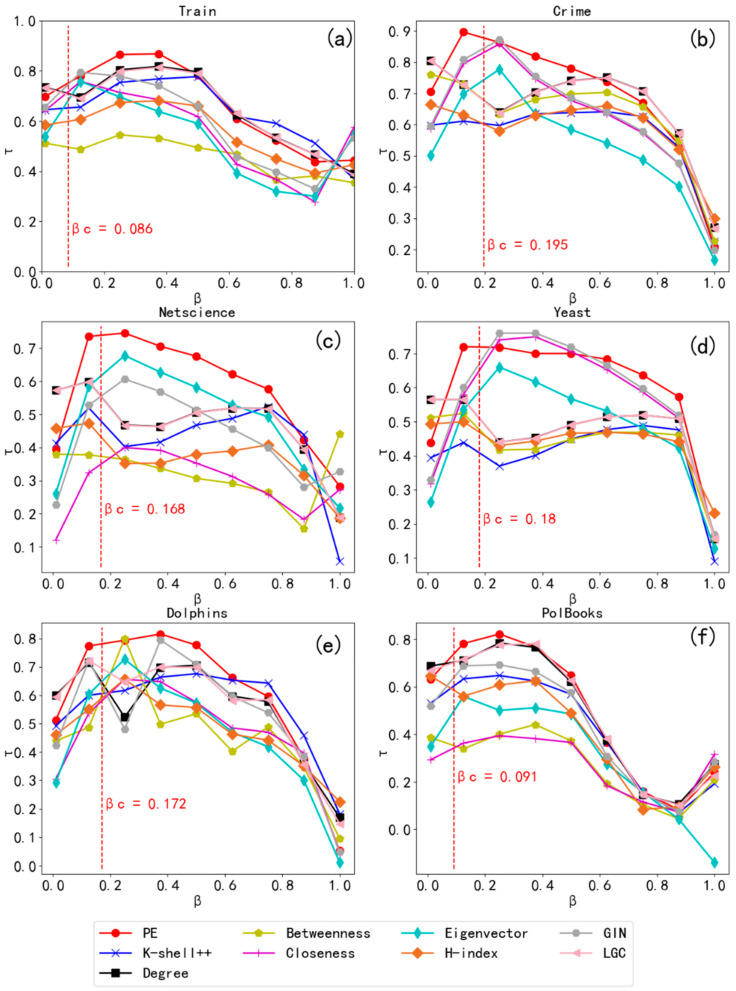
Performance comparison of the critical indicators in the SIRS model for different values of *β* (infection rate). Each subgraph (**a**–**f**) of the graph represents a real network; the network name is at the top of the graph, the vertical dashed line inside the graph indicates the prevalence threshold of that network, and the performance of the node *PE* metric is highlighted in red. The higher the *τ* value, the better the performance of the corresponding centrality methods.

The correlation between the rankings lists provided by nine different importance measures, six real networks in different domains, and the rankings lists obtained from the SIRS model by adjusting the infection rate β are shown in Figure 5.

Figure 5 shows that *PE* performed the best compared to the other eight importance measures on both small and large networks. We found that the performance of degree centrality was best when the value of *β* was small. This might be because when the infection rate is small, it is difficult for infected nodes to infect other nodes; at this time, the more neighboring nodes a node has, the more likely it is that the node will infect other nodes, which is consistent with reality. Figure 5 shows that the rankings list obtained from *PE* correlates more strongly with that obtained from the SIRS model as the infection rate increased, especially when *β* was near the prevalence threshold, and was higher than other importance indicators. Therefore, the rankings list of nodes obtained by *PE* was more accurate, and *PE* identified important nodes in the network accurately.

Table 4 shows that we obtained the *τ* values of each importance metric under the prevalence threshold of each network, from which it can be seen that the node *PE* metric performed better than the other importance metrics; *PE* performed the best on all six networks.

Figure 5 and Table 4 show that in the six real networks used in the SIRS model experiment, regardless of size, the importance rankings list obtained by *PE* was more closely related to the real importance rankings list simulated by the SIRS model. Furthermore, the *τ* value was higher and better than that of the other eight importance indexes. In addition, the results show that *PE* could identify the important nodes in the network in the SIRS model.

### 4.6. Robustness Experiment

This section evaluates the accuracy of the algorithm in identifying important nodes from the perspective of robustness [42] and whether the significance of a node is determined by the impact on network connectivity after removing the node. We assess the impact of node failure on network connectivity through the largest connectivity coefficient [43]; the greater the impact, the more important the failed node is.

A connected component is a network subgraph in which any two nodes in a subgraph are connected. There are many disconnected networks in the real world. These are broken down into multiple connected components, in which the connected component with the largest number of nodes is called the largest connected component [43]. The size of the largest connected component reflects the connectivity of a complex networks. The scale of the largest connected component changes owing to the removal of nodes. After removing the important nodes, its scale becomes smaller; the greater the change, the more crucial the nodes that were removed. Hence, the robustness of the network was estimated with the largest connectivity coefficient.

The largest connected coefficient (denoted *r*) can be defined as the ratio of the number of nodes contained in the network’s largest connected component to the overall number of nodes in the network. It is formulated as follows:

(19) $$r=\frac{n_{c}}{n}$$

where *n_c_* denotes the number of nodes contained in the network’s largest connected component after the removal of some nodes and *n* denotes the total number of nodes in the network. The value varies according to the ratio of the number of nodes removed from the network to the overall number of nodes in the network, which is denoted *f*. A gradual decrease in the *r* value is observed as the number of nodes removed increases.

**Figure 6:**
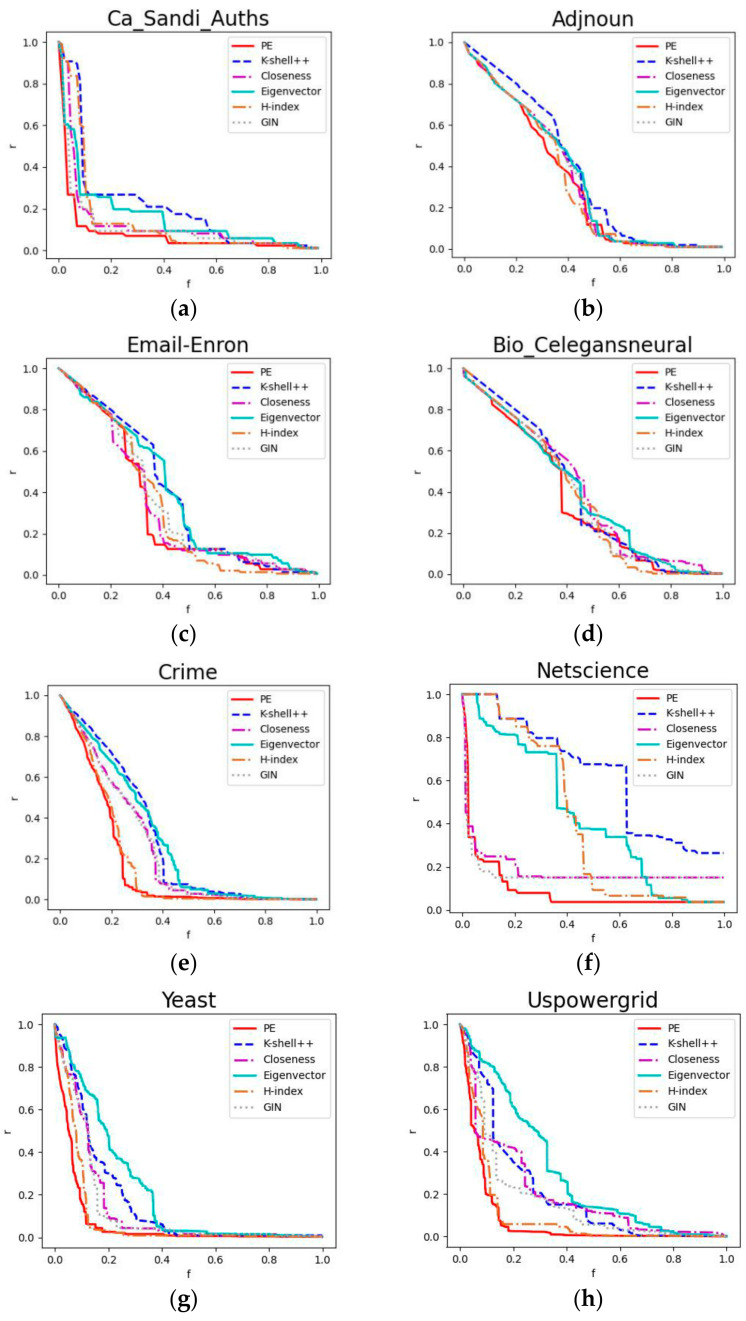
Downward trend of the maximum connectivity coefficient of the critical indicators for different values of *f* (the proportion of removed nodes to the overall number of nodes). The horizontal coordinate denotes the node removal ratio *f*, and the vertical coordinate denotes the largest connectivity coefficient value, *r*, after the removal of the node. Each subgraph (**a**–**h**) of the graph demonstrates the results of an experiment on a real network. The subgraph is ordered in increments by the total number of network nodes. At the same node removal scale, *f*, the more noticeable the maximum connectivity coefficient downtrend is, the more important the removed node is deemed to be. The graphs show that the downward trend of *PE* on eight networks is faster than that of the other five indicators, illustrating that *PE* performed the best. In conclusion, the *PE* indicator has the highest practical value.

By drawing the network nodes on two-dimensional coordinates in terms of the importance evaluation algorithm, the curve of the change in the largest connectivity coefficient of the network was analyzed after the nodes were removed one by one on the basis of their order of importance, from largest to smallest. The more pronounced the downward trend of the curve, the better the effect of the algorithm. Eight different real-world networks respectively used six different importance measures to sort the nodes and remove them in order of importance from largest to smallest (Figure 6).

We used the robustness value, *R* [42,44], to estimate the performance of the method; *R* is calculated as follows:

(20) $$R=\frac{1}{n}\sum_{j=1}^{n}r_{j}$$

where *n* denotes the total number of nodes in the original network and *r_j_* signifies the largest connected coefficients after removing *j* nodes. Every time a node is removed, the largest connectivity coefficient of the network is calculated and added to *R*; this process iterates until the network is empty. Consequently, the smaller the final *R* value is, the faster the network crashes, illustrating that the important nodes identified by the algorithm are more accurate.

We analyzed the robustness of the above six different importance measures on the basis of connectivity. Table 4 presents the evaluation results of their robustness, *R*.

From Equation (19), it was inferred that the smaller the robustness value, the faster the network collapses, expressed as better performance of the algorithm in identifying important nodes. Table 5 shows that *PE* rapidly reduced the maximum connectivity coefficient, *r*, of the network on all eight networks, with the smallest *R* value, and identified the important nodes in the network. The results verify that when *PE* removed network nodes in order of importance from largest to smallest, it minimized the robustness; i.e., the node *PE* metric accurately identified the important nodes in the network.

## 5. Discussion

This paper proposed a novel importance metric, node *PE*, for identifying critical nodes in complex networks. Node *PE* achieves this by combining the effects of the clustering coefficients and number of first- and second-order neighbor on the importance of nodes from an entropy perspective while considering the global and local information of the network. We applied the proposed method to nine real networks in order to evaluate its performance, and simulated the propagation process using the epidemic model. The ranking correlation between the rankings lists (those generated by different centrality metrics and that generated by simulation results) was measured using the Kendall coefficient, *τ*.

The results of comparative experiments conducted using nine different importance measures, specifically, K-Shell++, DC, BC, CC, EC, H-index, GIN, and LGC, showed that our proposed node *PE* metric provides superior performance and is relatively stable as compared to other methods. We observed that *PE* provided very accurate results in the epidemic models as well as in the robustness experiments. Furthermore, our proposed measure outperformed other measures and was consistent on most networks. Through experimental demonstrations on twelve real networks from different domains, the proposed node *PE* metric proved to be more effective and stable in identifying significant nodes in complex networks. However, there are limitations to node *PE*, such as the fact that it is designed to be used only on undirected networks. In future work, we will investigate the viability of the node *PE* metric in directed networks and verify whether *PE* is predictive in real-world contexts.

## Figures and Tables

**Table 1:** Accuracy comparison of important proteins identified by ten different centrality indicators.

Network	PE	K − Shell + +	DC	BC	CC	EC	VoteRank	H-Index	GIN	LGC
Saccharomyces	0.375	0.404	0.402	0.35	0.354	0.367	0.351	0.405	0.364	0.404

**Table 2:** Topological features of the twelve actual networks used in the study.

Network	*n*	*m*	<*c*>	<*k*>	*d*
AdjNoun	112	425	0.173	7.589	0.068
Train	64	243	0.561	7.593	0.120
Karate	34	78	0.255	4.588	0.139
Ca_Sandi_Auth	86	124	0.414	2.883	0.034
Email-Enron	143	623	0.434	8.713	0.061
Dolphins	62	159	0.308	5.129	0.084
Polbooks	105	441	0.348	7.589	0.068
Bio_celegansneural	297	2300	0.311	15	0.053
Crime	1380	1476	0.009	2.14	0.002
Yeast	1870	2277	0.094	2.435	0.001
Netscience	1461	2742	0.694	3.753	0.001
Uspowergrid	4941	6594	0.08	2.669	0.001

Note: *n*, total number of nodes in each network; *m*, total number of connected edges in each network; $\langle c\rangle =\frac{\sum_{i}^{n}c_{i}}{n}$ is the average clustering coefficient of each network; $\langle k\rangle =\frac{\sum_{i}^{n}k_{i}}{n}$ is the average degree of each network; and $d=\frac{2m}{n\left(n-1\right)}$ is the network density.

**Table 3:** Performance of the SIR model for ten indicators under a prevalence threshold, β, of nine networks. The best-performing results are highlighted in bold.

Network	PE	K − Shell + +	DC	BC	C	EC	PE(N2)	H-Index	GIN	LGC
AdjNoun	0.894	0.780	0.828	0.641	0.866	**0.925**	0.844	0.716	0.905	0.827
Train	**0.892**	0.740	0.830	0.558	0.763	0.761	0.849	0.661	0.857	0.809
Ca_Sandi_Auth	**0.858**	0.555	0.572	0.428	0.658	0.772	0.808	0.433	0.776	0.578
Email − Enron	**0.854**	0.768	0.822	0.488	0.676	0.853	0.844	0.703	0.852	0.827
Dolphins	**0.909**	0.713	0.747	0.541	0.652	0.707	0.898	0.577	0.856	0.75
Polbooks	**0.864**	0.715	0.762	0.362	0.378	0.601	0.802	0.611	0.737	0.775
Crime	**0.869**	0.596	0.648	0.654	0.854	0.786	0.857	0.582	0.861	0.647
Netscience	**0.751**	0.471	0.540	0.378	0.337	0.656	0.730	0.424	0.551	0.539
Yeast	**0.748**	0.407	0.503	0.474	0.672	0.606	0.689	0.460	0.694	0.503

**Table 4:** Performance of the SIRS model for nine indicators under the prevalence threshold, β, of nine networks. The best-performing results are highlighted in bold.

Network	PE	K − Shell + +	DC	BC	CC	EC	H-Index	GIN	LGC
Train	**0.770**	0.676	0.722	0.477	0.718	0.696	0.635	0.762	0.716
Dolphins	**0.802**	0.646	0.691	0.497	0.596	0.672	0.525	0.768	0.688
Polbooks	**0.745**	0.641	0.676	0.341	0.352	0.550	0.522	0.670	0.671
Crime	**0.875**	0.592	0.649	0.651	0.853	0.775	0.582	0.868	0.649
Netscience	**0.744**	0.473	0.552	0.378	0.341	0.644	0.431	0.549	0.551
Yeast	**0.747**	0.400	0.495	0.468	0.668	0.610	0.473	0.692	0.495

**Table 5:** Robustness (*R*) values for six indicators of eight networks. The best-performing results are highlighted in bold.

Network.	PE	K − Shell + +	CC	EC	H − Index	GIN
Ca_Sandi_Auth	**0.082**	0.210	0.131	0.164	0.143	0.112
AdjNoun	**0.315**	0.373	0.329	0.338	0.316	0.333
Email − Enron	**0.319**	0.386	0.325	0.391	0.324	0.347
Bio_celegansneural	**0.351**	0.387	0.398	0.389	0.364	0.374
Crime	**0.168**	0.291	0.246	0.290	0.180	0.247
Netscience	**0.093**	0.625	0.186	0.434	0.408	0.172
Yeast	**0.065**	0.163	0.132	0.213	0.084	0.123
Uspowergrid	**0.073**	0.200	0.197	0.294	0.097	0.165

## Data Availability

The datasets generated during and analyzed during the current study are available in The Network Data Repository, https://networkrepository.com, accessed on 13 December 2021.
